# Investigating the Influence of Role-Playing on Empathy and Perspective-Taking by Analyzing Level of Engagement, Emotional, and Cognitive Processes Through a Word Count Analysis Approach

**DOI:** 10.5334/pme.1482

**Published:** 2025-11-10

**Authors:** Yi-Min Tien, Chia-Yao Lin, Pei-Ying Pai, Chon-Haw Tsai, Li-Chuan Hsu

**Affiliations:** 1 Department of Psychology, Chung Shan Medical University, Taichung, Taiwan; 2 Clinical Psychological Room, Chung Shan Medical University Hospital, Taichung, Taiwan; 3 School of Medicine, China Medical University, Taichung, Taiwan; 4 Department of Internal Medicine, Division of Cardiology, China Medical University Hospital, Taichung, Taiwan; 5 Graduate Institute of Biomedical Sciences, China Medical University, Taichung, Taiwan; 6 Department of Neurology, China Medical University Hospital, Taichung, Taiwan; 7 Neuroscience Laboratory, China Medical University Hospital, Taichung, Taiwan

## Abstract

**Background:**

Role-playing is widely used to foster empathy and perspective-taking in medical education, but the underlying psychological mechanisms remain unclear. This study investigated whether actual role enactment enhances engagement compared to imagination alone, and how immersive role-play influences students’ emotional and cognitive processes.

**Methods:**

Sixty second-year medical students participated in a two-stage intervention: the Imagination Stage (imagining being a patient or caregiver) and the Role-Playing Stage (actively enacting both roles in a counterbalanced design). After each stage, students wrote reflections. Language use was analyzed using the Chinese version of the Linguistic Inquiry and Word Count (LIWC) tool. Function word frequency was used to calculate the Level of Engagement (LOE). Along with affective and cognitive word usage, these measures provided insights into students’ psychological processes. Human ratings of empathy and perspective-taking were conducted by trained psychology students.

**Results:**

Reflections from the Role-Playing Stage—particularly in the caregiver role—showed significantly higher LOE compared with the Imagination Stage. Higher LOE scores correlated with increased human-rated empathy and perspective-taking. LIWC analysis revealed that patients used more first-person pronouns and negative emotion words, while caregivers used more third-person pronouns, conjunctions, causation, and discrepancy words. Role-playing also led to a decrease in first-person pronouns, negative emotion words, and anxiety words, with an increase in conjunctions and certainty words.

**Conclusion:**

Immersive role-play enhances students’ emotional and cognitive engagement beyond imagined scenarios. Objective linguistic analysis provides valuable insights into learners’ psychological processes, highlighting the pedagogical value of immersive role-play in fostering empathy and perspective-taking in medical education.

## Introduction

Empathy and perspective-taking are fundamental for effective communication in healthcare settings [1]. Empathy allows healthcare professionals to understand and share patients’ emotions and experiences, fostering deeper connections and trust. These bonds, in turn, create a supportive environment where patients feel respected and valued [2,3]. Perspective-taking complements this process by helping providers comprehend patients’ thoughts, beliefs, and concerns, enabling more personalized, patient-centered care [4]. Together, empathy and perspective-taking are critical to building strong patient-provider relationships and enhancing diagnostic accuracy, treatment planning, and collaborative decision-making [2,5].

In medical education, empathy is widely conceptualized as comprising both affective and cognitive components [6,7]. The affective dimension pertains to the emotional resonance between healthcare professionals and their patients, while the cognitive dimension involves accurately understanding and communicating patients’ emotional states [8]. To nurture these skills in future healthcare providers, undergraduate medical programs often incorporate role-playing exercises and clinical simulation training [9,10], aiming to promote both emotional engagement and cognitive understanding.

Role-playing, in particular, provides an immersive learning experience grounded in experiential learning theory [11,12,13]. By adopting the roles of patients or caregivers, students can experience emotions such as fear or vulnerability from the patient’s perspective, thereby deepening their understanding [9,14,15]. Furthermore, role-playing supports cognitive development by enhancing comprehension, critical thinking, and problem-solving skills. It is especially valuable in caregiver roles, where learners must refine communication strategies and adapt flexibly to diverse situations through repeated practice [16].

Despite these documented benefits, the current literature lacks a comprehensive understanding of the psychological processes underlying role-playing’s impact. Most studies have relied on subjective methods such as pre- and post-intervention questionnaires [14], self-reflective reports [17], observer assessments [18], and satisfaction surveys [19], which may not fully capture students’ internal engagement. Moreover, while role-playing demands full alignment with the character’s perspective—requiring participants to act and speak consistently with their assigned roles [20]—little research has objectively compared actual role enactment with mere role imagining. The emotional and cognitive mechanisms involved also remain underexplored. In addition, the broader literature has raised concerns about the effectiveness of role-based simulations—including patient, caregiver, and even disability simulations—arguing that such practices may not consistently produce authentic or long-term improvements in empathy or perspective-taking [21,22,23].

To address these gaps, we employed the Linguistic Inquiry and Word Count (LIWC) [24] tool to analyze students’ reflective writing during role-playing activities, aiming to gain insight into their psychological processes. LIWC calculates the frequency of various word categories—including function words, affective words, and cognitive words—thereby offering both structural and content-related information about language use. This analysis enables the identification of ***language styles*** that reflect emotional, cognitive, and interpersonal states [25,26].

Specifically, affective words can reveal students’ emotional engagement [27], while cognitive words indicate reflection, reasoning, and meaning-making [26]. These linguistic dimensions are particularly salient in role-play contexts, where students are tasked with adopting unfamiliar perspectives and navigating emotionally charged interactions. A growing body of research supports the psychological relevance of LIWC’s word categories, demonstrating their applicability in educational and clinical settings [28,29].

In studying empathy and perspective-taking—two critical elements of medical professionalism—certain linguistic markers offer particularly valuable insights. Research has shown that the use of personal pronouns (e.g., “I,” “you”) can indicate a shift in viewpoint, facilitating greater self–other awareness and empathetic engagement [30,31]. Additionally, auxiliary verbs such as “am” and “will” can convey emotional depth and immediacy, fostering emotional engagement [32]. The appearance of negative emotion words may reflect students’ emotional involvement or express a compassionate response to the simulated suffering of others [33]. Analyzing these linguistic markers with LIWC allows us to trace how students linguistically express and develop empathy and perspective-taking throughout the course of the role-play.

Beyond analyzing individual word categories, we also examined the overall stylistic alignment between students’ writing and their assigned roles using the metric of Language Style Synchrony (LSS), developed by Ireland and Pennebaker [34]. LSS measures the degree to which communicators coordinate their use of function words, which has been interpreted as reflecting psychological alignment and emotional attunement. Notably, Lord et al. [35] demonstrated that higher LSS scores—even when calculated from the therapist’s speech alone—were predictive of greater perceived empathy. This synchrony in function word usage suggests a deeper cognitive concordance and affective resonance, both of which are hallmarks of empathetic communication.

Taken together, LIWC and LSS offer a multidimensional linguistic approach to understanding students’ emotional and cognitive engagement during role-playing. These tools allow for a nuanced analysis of internal learning processes and support the development of empathy and professional identity—an area of growing importance in medical education.

### Main purpose

Our study investigated whether immersive role-playing enhances empathy and perspective-taking in medical students. We defined immersion, or Level of Engagement (LOE), as the degree to which participants synchronized with the characters they portrayed—acting and speaking consistently within those roles. This synchronization reflects how well the portrayed behaviors in a communication interaction are coordinated in terms of timing and form, rather than being random or disjointed. Using Language Style Synchrony (LSS) [34,35], we measured LOE as a proxy for role-playing engagement. To compare imagined versus enacted engagement, we analyzed students’ reflective writing from two phases: the Imagination Stage, where students only imagined playing the roles of patients or caregivers, and the Role-Playing Stage, following actual role enactment. We aimed to determine whether the effectiveness of role-playing in student learning requires actual participation and immersion in the roles, or if simply imagining the scenario without active engagement would suffice.

LIWC was employed to examine shifts in function, emotional, and cognitive word usage across both stages and roles. We hypothesized that greater role immersion, as indicated by higher LOE and richer emotional/cognitive language, would reflect enhanced empathy and perspective-taking. This approach sought to offer a more objective understanding of how role-playing contributes to humanistic learning in medical education.

## Methods

### Subjects

This study recruited 60 second-year medical students (35 males, 25 females; Mean age = 20.5) from China Medical University (2018–2019), all of whom voluntarily enrolled in an elective course. The number of participants was limited by the course capacity, as the nurse-teaching simulation room used for role-playing could only accommodate 20 students per semester. Therefore, data from three semesters were combined to reach a total of 60 participants. To ensure a similar baseline, eligibility was restricted to second-year students who had not received prior formal training in patient care or role-playing.

### Study design and procedure

#### Procedure of Role-Play Sessions

Role-playing was a core component of a three-week module within the elective course “Communication: Theory and Practice,” designed for medical students to provide hands-on experience in developing empathy, perspective-taking, and communication skills. The primary objectives of this activity were threefold: enhancing emotional empathy by immersing students in the patient’s experience, developing perspective-taking skills through switching roles between patient and caregiver, and encouraging self-reflection via structured reflective writing. Before enrollment, students could review the course website, which clearly outlined these objectives.

This design prioritized human interactions over clinical decision-making, avoiding reliance on students’ prior medical knowledge. Therefore, the roles were limited to patient and caregiver, rather than patient and doctor. To ensure safety, caregivers continuously assisted patients in a controlled environment. All participants provided informed consent before the study, ensuring that they fully understood the tasks and potential risks, and retained the right to withdraw at any time. The study was approved and supported by the Teaching Practice Research Program in the Ministry of Education in Taiwan (PMN107098). The LIWC procedure was approved by the Institutional Review Board (CS19134).

**Figure 1:**
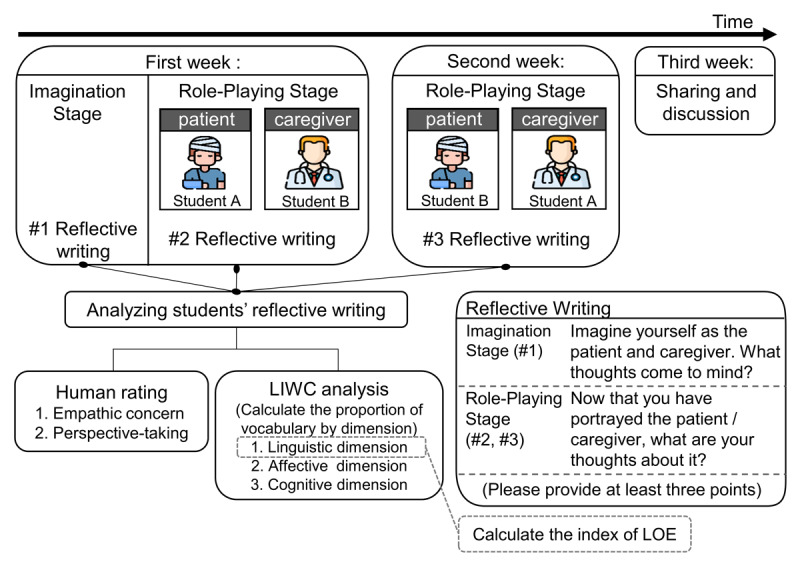
Design of the Role-Play Activity. This figure illustrates a three-week role-play activity in which students alternated between the roles of patient and caregiver. Reflective writing was conducted at three points: during the Imagination Stage, after the first week of role-play, and after switching roles in the second week. In the third week, students participated in a sharing and discussion session. Reflections were analyzed using human ratings (empathy and perspective-taking) and LIWC analysis (linguistic, affective, and cognitive dimensions) to assess learning outcomes and engagement.

Each semester, the 20 students were randomly paired into 10 groups, and the role-playing activities took place in a nurse-teaching simulation room equipped with 10 hospital beds. The activity was structured into two main stages throughout the first two weeks: the Imagination Stage and the Role-Playing Stage. The full process is illustrated in Figure 1.

In Week 1, students began with the Imagination Stage. They spent five minutes imagining themselves in the role of a patient, followed by five minutes imagining the caregiver’s role. They then recorded their thoughts and feelings in response to the first two questions on a reflective writing sheet (#1 reflective writing). After this, students were randomly assigned to either the patient or caregiver role. For the Role-Playing Stage, caregivers assisted patients by blindfolding them and securely binding their legs together to simulate a physical disability. Patients lay in bed while caregivers identified their needs and provided care (see Supplementary Table 1). The session lasted 90 minutes. Afterward, students reflected on the experience by responding to a third or fourth question on the writing sheet (#2 reflective writing).

In Week 2, Students alternated roles in a counterbalanced design, ensuring everyone experienced both perspectives. This approach minimized potential role order effects and facilitated a more balanced comparison between the imagination and enactment stages. They repeated the same procedures and completed a final round of reflection (#3 reflective writing). In Week 3, the teacher showed video recordings of previous sessions for a collective review and discussion of their experiences. Participant safety was prioritized throughout the role-play. All students were informed that they could withdraw from the simulation at any time if they experienced discomfort. In addition, those who preferred not to simulate disability were allowed to conclude the activity beforehand, with caregivers providing standard assistance. These safeguards ensured the dignity and well-being of all participants.

### Human rating

To ensure the accuracy and consistency of the role-play activity evaluation, we first provided training for 15 senior students from the Department of Psychology at Chung Shan Medical University. These students were qualified to serve as raters, as they had completed coursework in counseling and communication psychology and had experience with client case discussions.

Before the formal rating process, all raters participated in three consensus meetings. During these sessions, the criteria for empathic concern and perspective-taking, as defined by Davis [36], were thoroughly explained and discussed to help raters understand how to apply these standards effectively. The meetings also involved reviewing and evaluating sample reflections together. By comparing rating outcomes and resolving discrepancies through discussion, the raters collaboratively established consistent rating guidelines. These consensus meetings facilitated a shared understanding of the evaluation criteria among raters, thereby ensuring greater reliability and consistency in their subsequent assessments.

Empathic concern was assessed based on whether role-players demonstrated compassion and emotional engagement in their assigned roles. For example, when portraying a patient, did the student show empathy toward the character? As a caregiver, did the student express care and sympathy towards the patient, particularly in difficult situations? Perspective-taking was evaluated based on the students’ ability to adopt different viewpoints and respond from another’s perspective. For instance, could the student immerse themselves in the patient’s role and express thoughts and feelings from that viewpoint? Similarly, as a caregiver, did the student reflect and respond from the caregiver’s perspective?

All ratings were conducted using a 7-point Likert-type scale to quantify the degree of empathy demonstrated during the role-play. For data analysis, we used descriptive statistics and conducted two t-tests: one to compare differences in empathic concern between the Imagination and Role-Playing stages, and another to assess changes in perspective-taking across the two stages.

### LIWC analysis

The LIWC program classifies words into 64 nonexclusive categories. For our analysis, we focused on three key dimensions—linguistic, affective, and cognitive—each comprising well-established LIWC categories. Specifically, the linguistic dimension includes 11 categories of function words, while the affective and cognitive dimensions include 5 and 6 categories, respectively [24,25,26]. We used the Chinese version of LIWC [37], and the percentage of words in each category served as the dependent variable (see Supplementary Table 2). Since articles do not exist in the Chinese language, they were not included in the analysis.

Our statistical analysis involved two main steps. First, we calculated the Level of Engagement (LOE)—an indicator of immersion in the role-playing sessions—based on the aggregate frequency of the 11 linguistic categories [34,35,38]. LOE was treated as a continuous variable ranging from 0 to 1, with higher values reflecting greater use of linguistic features and, by extension, deeper engagement. LOE scores were computed separately for the Imagination Stage and the Role-Playing Stage. A mixed-design two-way repeated-measures ANOVA was then conducted to investigate the effects of Stage (within-subjects: Imagination vs. Role-Playing) and Role (between-subjects: patient vs. caregiver) on LOE. In addition, we examined the relationship between LOE and human-rated empathy and perspective-taking using Pearson’s correlations.

Second, to investigate changes in emotional and cognitive processes, we analyzed word usage across the linguistic, affective, and cognitive dimensions. Since multiple word categories were assessed simultaneously within each dimension, we employed MANOVA for a more comprehensive analysis. Three two-way repeated-measures MANOVAs were conducted, with Stage as a within-subjects factor and Role as a between-subjects factor. Word categories within each dimension were treated as multiple dependent variables. Significant effects were further interpreted using univariate F-tests.

## Results

### Results of human rating

To ensure the reliability of ratings, we calculated inter-rater reliability using Intraclass Correlation Coefficient (ICC) [39], the calculated ICC was 0.35 (*p* < 0.0001), indicating a moderate level of agreement among raters.

Human ratings showed significant increases in both empathic concern and perspective-taking during the Role-Playing Stage. Empathic concern rose from 3.51 (SE = 0.29) to 3.94 (SE = 0.27), *t*(14) = 2.33, *p* < 0.05, Cohens’*d* = 0.40; perspective-taking increased from 4.11 (SE = 0.31) to 4.36 (SE = 0.32), *t*(14) = 3.05, *p* < 0.01, Cohens’*d* = 0.23.

### Results of the LIWC analysis

#### Step 1: Level of Engagement (LOE)

**Figure 2:**
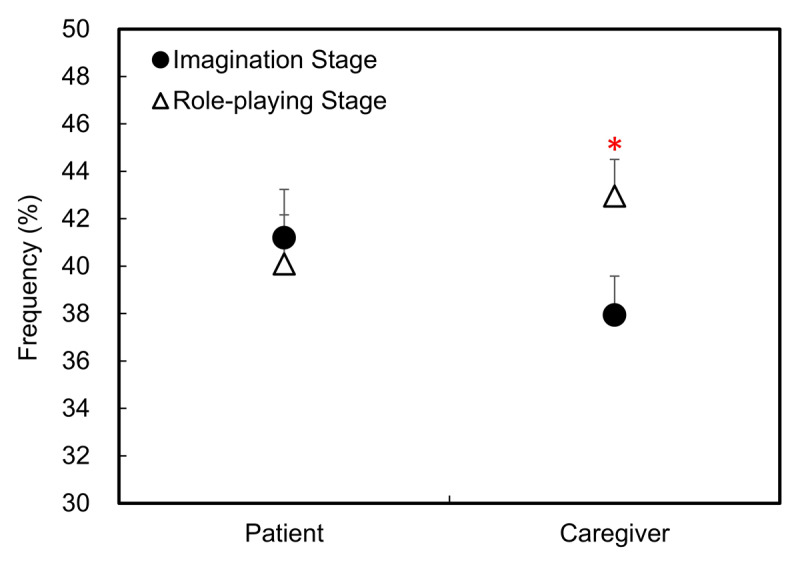
The results of the Level of Engagement are presented. The y-axis indicates the average frequency of all word categories in the linguistic dimension. Black circles indicate word usage in the Imagination Stage, with open triangles showing those in the Role-Playing Stage. Error bars represent 0.5 standard error. An asterisk (*) indicates *p* < .05.

There were no significant main effects of Role or Stage (*p*s > 0.1), but a marginally significant interaction emerged, *F*(1, 59) = 3.87, *p* = 0.054, *η*^2^ = 0.06. Planned contrasts showed that caregivers demonstrated higher LOE during the Role-Playing Stage than in the Imagination Stage, *t*(59) = 2.15, *p* < 0.05, Cohens’*d* = 0.36 (see Figure 2). This suggests that students used a more immersive and engaged linguistic style when actively embodying the caregiver role.

**Correlation between the LOE and human rating:** We used Pearson’s correlations to examine the link between LOE and empathy ratings across both stages. Only the caregiver role in the Role-Playing Stage showed significant correlations between LOE and both empathy (r = 0.27, *p* < 0.05) and perspective-taking (r = 0.35, *p* < 0.05). No other significant correlations were found (see Supplementary Table 3).

#### Step 2: Two-way repeated-measures MANOVA (see Supplementary Table 4)

**Figure 3:**
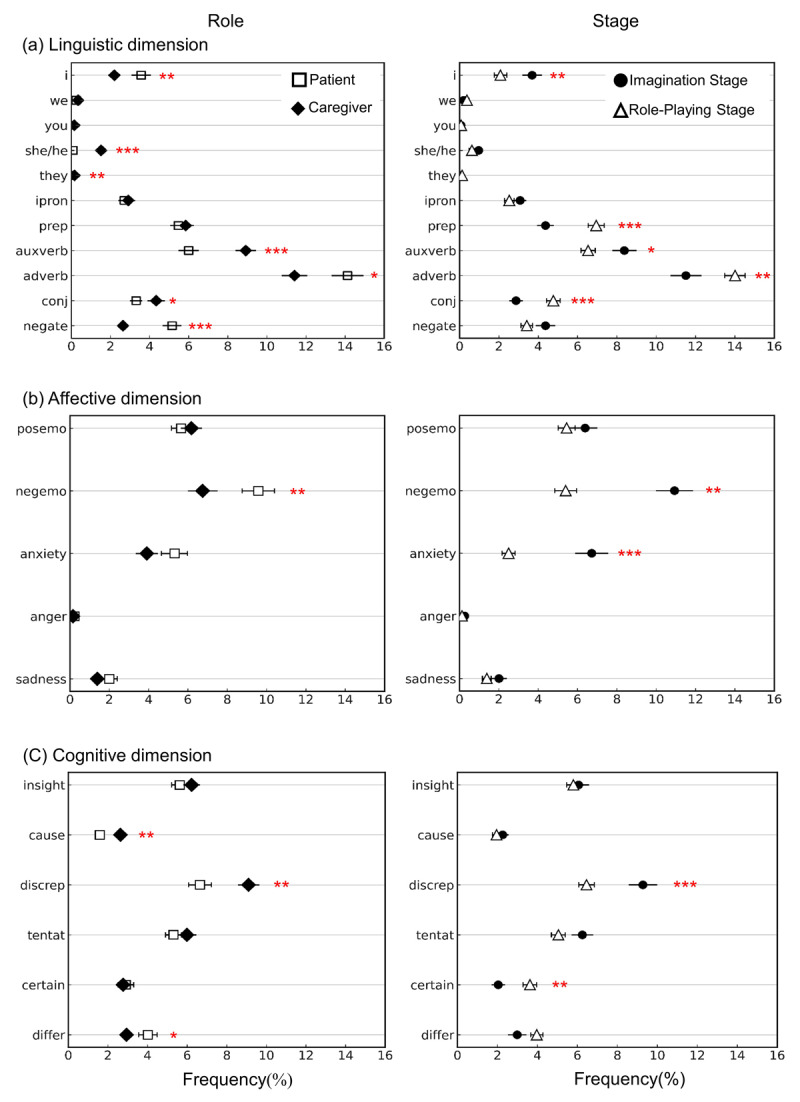
Results of the word count analysis for each word category across the linguistic, affective, and cognitive dimensions. The y-axis represents the frequency of each word category. Open squares represent students role-playing as patients, and black diamonds represent students role-playing as caregivers. Black circles indicate word usage during the Imagination Stage, while open triangles indicate word usage during the Role-Playing Stage. **(a)** ipron = impersonal pronouns; prep = prepositions; auxverb = auxiliary verbs; adverb = common adverbs; conj = conjunctions; negate = negations. **(b)** posemo = positive emotion; negemo = negative emotion. (c) cause = causation; discrep = discrepancy; tentat = tentative; certain = certainty; differ = differentiation. Error bars represent 0.5 standard error. One asterisk (*) indicates p < .05, two asterisks (**) indicate p < .01, and three asterisks (***) indicate p < .001.

**Linguistic dimension:** Both Role and Stage had significant main effects, as indicated by Wilk’s Λ = 0.25, *F*(11, 49) = 13.32, *p* < 0.001, *η*^2^ = 0.75, and Wilk’s Λ = 0.41, *F*(11, 49) = 6.53, *p* < 0.001, *η*^2^ = 0.59, respectively. However, their interaction was not significant (*p* > 0.1) (See Figure 3(a)).

F-tests were conducted to examine Role effects on word categories. Results showed that students used “I,” adverbs, and negations significantly more in the patient role than in the caregiver role (all *F*(1, 59) > 6.94, *p* < 0.05, *η*^2^ > 0.11). Conversely, in the caregiver role, they used “she/he,” “they,” auxiliary verbs, and conjunctions more frequently than in the patient role (all *F*(1, 59) > 3.91, *p* < 0.05, *η*^2^ > 0.06). One student acting as a caregiver wrote:

- *Hope* ***he would*_(auxiliary verbs)_** *feel comfortable during role-playing. The campus needs more accessible space for blind and handicapped people*.

The Stage effects showed that in the Imagination Stage, students used “I” and auxiliary verbs more frequently than in the Role-Playing Stage (both *F*(1, 59) > 6.97, *p* < 0.05, *η*^2^ > 0.11). As for the using of “I”, this is evident in two statements made by students in the Imagination Stage:

- ***I** am nervous about acting as a patient*.
- ***I** am worried about the role-playing event, and **I** may get hurt*.

Conversely, in the Role-Playing Stage, they used prepositions (pre), adverbs (adv), and conjunctions (con) more frequently than those in the Imagination Stage, with all *F*s(1, 59) > 8.90, *p*s < 0.01, *η*^2^ > 0.13. For example, one student wrote prepositions, adverbs, and conjunctions:

- ***When*_(con)_** *I helped him* ***cross*_(pre)_** *the road, I had* ***to*_(pre)_** *watch* ***out*_(adv)_ *for*_(pre)_** *cars* ***and*_(con)_** *ensure the ground was even, emphasizing that crossing the road is* ***very*_(adv)_** *dangerous*, ***and*_(con)_** *he must follow my instructions* ***to*_(pre)_** *cross* ***safely*_(adv)_**.

Two students acting as caregivers wrote:

- *I wanted* ***to*_(pre)_** *help*, ***but*_(con)_** *I realized I couldn’t* ***fully*_(adv)_** *replace someone’s eyes*.
- *Guiding someone is harder* ***than*_(con)_** *I thought; I kept assuming they could do things they* ***actually*_(adv)_** *couldn’t*.

**Affective dimension:** Results showed a significant main effect for Stage, with Wilk’s Λ = 0.60, *F*(5, 55) = 7.31, *p* < 0.001, *η*^2^ = 0.4. No significant main effect for Role or interaction effect for Role and Stage were observed (*ps* > 0.1) (See Figure 3(b)).

To examine the Role effect, an F-test was conducted. The results showed that students used more negative emotion words in the patient role than in the caregiver role, *F*(1, 59) = 8.07, *p* < 0.01, *η*^2^ > 0.12. For example, one student, acting as a patient in the Imagination Stage, wrote:

- *I was **afraid** to fall down because I could not see*.

The analysis did not show a statistical difference in the use of positive emotion words between roles, but some examples of positive emotion words expressed by students are provided here. For instance, a student playing the patient role wrote:

- As a patient, it is important to constantly ***communicate*** your needs to the caregiver.
- I am ***grateful*** to the caregiver, who was very patient.
- Having someone ***accompany*** you provides great ***support***, both in daily life and emotionally.
- *I’m* ***grateful*** *I can still see*.

However, there were also students acting as caregivers who experienced negative emotions after the role-playing. Another two students, acting as caregivers, wrote:

- *He was too heavy for me to move the wheelchair, so I felt **helpless.***
- *Taking care of patients is very **strenuous**, and I felt **guilty** that I couldn’t play the role well*.

Regarding the Stage effects, the F-tests revealed that in the Imagination Stage, students used more negative emotion and anxiety words compared to the Role-Playing Stage, with both *Fs*(1, 59) > 24.71, *ps* < 0.001, *η*^2^ > 0.30 (See Figure 3(b)). For example, one student imagined acting as a patient and wrote the following:

- *I may get **hurt***.
- *This is my first time joining this kind of class. I am **worried** about playing the blind patient*.

Another student, imagining acting as a caregiver, wrote:

- *I am **afraid** I will put him/her in danger*.

**Cognitive dimension:** Both Role and Stage had significant main effects, with Wilk’s Λ = 0.75, *F*(6, 54) = 2.96, *p* < 0.05, *η*^2^ = 0.25, and Wilk’s Λ = 0.71, *F*(6, 54) = 3.66, *p* < 0.01, *η*^2^ = 0.29, respectively. No significant interaction effect was found for Role and Stage (*ps* > 0.1).

F-tests showed that students used more causation and discrepancy words when playing the caregiver role than the patient role, *F*s(1, 59) > 5.97, *p*s < 0.05, *η*^2^ > 0.09. In an example of causation word usage, when acting as a caregiver, one student wrote:

- *We should avoid areas by the roadside where the surface is uneven and raised, **because** such conditions could potentially injure the patient*.

In an example of discrepancy word usage, when acting as a caregiver, one student wrote:

- *I **hope** he would feel better if the wheelchair would be placed that way*.

When students played the patient role, they used more differentiation words compared to when assuming the caregiver role, with *F*(1, 59) = 4.81, *p* < 0.05, *η*^2^ > 0.08. For example, when acting as a patient, one student wrote:

- *He encouraged me by saying it would be safe, **but** I still felt afraid*.

Regarding the Stage effects, F-tests showed that students used more discrepancy words in the Imagination Stage, *F*(1, 59) = 11.90, *p* < 0.001, *η*^2^ = 0.17, and more certainty words in the Role-Playing Stage, *F*(1, 59) = 10.43, *p* < 0.01, *η*^2^ = 0.15 (See Figure 3(c)).

- *I am **sure** that he and I would be very impressive in portraying roles during role-playing*.

These findings highlight the influence of both Role and Stage on linguistic, affective, and cognitive dimensions. Each dimension showed distinct patterns of word usage across the Role and Stage conditions.

## Discussion

Human ratings showed that participants’ empathy and perspective-taking improved when they engaged in the Role-Playing Stage. To assess engagement, we compared the Imagination and Role-Playing Stages using the LOE index. Results indicated that taking on the caregiver role led to greater use of function words in the Role-Playing Stage, reflecting higher LOE. This higher LOE was significantly correlated with empathy and perspective-taking ratings. Patients used more first-person pronouns and negative emotion words, while caregivers used more third-person pronouns, conjunctions, causation, and discrepancy words. In addition, the Role-Playing Stage showed reduced use of first-person, negative emotion, and anxiety words, and increased use of conjunctions and certainty words.

### Level of engagement reveals empathic communication

Our study compared students’ reflective writing between the Imagination Stage and the Role-Playing Stage. We found that the Level of Engagement (LOE) was significantly higher during the Role-Playing Stage, particularly for caregivers. This increase was closely tied to higher ratings of empathy and perspective-taking, reinforcing that empathic communication depends on the depth of role-playing engagement [37]. Role-playing in healthcare education requires full immersion between participants and the characters they portray, a process that can be traced through specific linguistic features.

Consistent with Lord et al. [35], we found that increased use of function words—such as pronouns, conjunctions, and auxiliary verbs—served as reliable indicators of empathy. For example, caregivers in the Role-Playing Stage used more function words like “and” and personal pronouns such as “he,” “she,” and “they,” indicating a conscious effort to construct more connected and emotionally nuanced communication with patients. In particular, our data also showed that students acting as caregivers used more auxiliary verbs (e.g., “am,” “will,” “have”) compared to those acting as patients, a finding that aligns with previous studies linking auxiliary verb use to higher empathy and perspective-taking scores in social media language [30,32]. These function words, including auxiliary verbs, add both functional and emotional depth to language, helping to create vivid, engaging narratives [30].

Our findings also align with the perception-action model [40], which suggests that perceiving another’s emotional state can activate a parallel state in the perceiver. When students deeply engage in role-playing, they tend to internalize the perspectives and emotions of their characters [35], leading to more empathetic and responsive communication. This behavioral alignment supports the development of empathy and perspective-taking [41], highlighting role-play as an effective tool for empathy training, particularly in healthcare education.

### Pronoun used by roles revealed perspective-taking

The use of pronouns in students’ reflective writing reveals how they adopted different perspectives during the role-playing process [42]. Students playing the patient role used more first-person pronouns (“I”), reflecting a self-focused viewpoint that may stem from the dependency embedded in the patient role. In contrast, those assuming the caregiver role used more third-person pronouns (“he,” “she,” “they”), indicating an outward focus and the ability to consider others’ experiences. While this result may initially seem intuitive, its implications extend beyond common sense. During reflective writing, students were free to express their thoughts using phrases such as “I think” or “In my opinion.” If students had remained primarily self-focused regardless of role, we would expect a high frequency of “I” across both groups. However, the findings revealed that caregiver-role students used more third-person pronouns, whereas patient-role students favored the first person. This suggests that those in the caregiver role genuinely adopted the perspective of another person, directing their attention toward the experiences and needs of the patient rather than simply reporting their own impressions.

These findings align with previous research suggesting that pronoun use reflects perspective-taking. For instance, Litvak et al. [30] found that frequent pronoun shifts on social media correlated with higher empathy, while Ricard et al. [43] demonstrated that children’s use of second- and third-person pronouns predicted their ability to take others’ perspectives. Our study extends these insights by showing how role-playing fosters both linguistic and cognitive shifts, enabling students to move beyond their own viewpoint and communicate with greater empathy.

### Experiencing emotional transformation

In the Imagination Stage, taking on the role of a patient elicited strong emotional responses among students, particularly negative emotions such as fear, helplessness, and discomfort. In our activity, students simulated being visually impaired and physically restricted for up to 90 minutes. These immersive experiences provided students with a more embodied understanding of the physical and psychological challenges faced by patients. Although the experience was uncomfortable, such emotional involvement laid a foundation for the development of empathy. This finding aligns with previous research showing that physicians with a history of illness tend to exhibit greater empathy and prioritize patient-centered care [44].

Interestingly, after engaging in the Role-Playing Stage, the proportion of negative emotion words in students’ reflections significantly declined. This suggests that through active participation, students developed a clearer understanding of the activity and anticipated fewer risks, leading to reduced expressions of negative emotion. Some students initially expressed fear of injury or causing harm but gradually shifted from personal discomfort toward a deeper understanding of patient needs and caregiving demands. For instance, one student expressed concern for their partner’s well-being and advocated for a more accessible campus environment—reflecting an emerging awareness of systemic barriers on campus (see Results, page 14). This movement from inward-focused unease to outward-directed concern illustrates a meaningful developmental trajectory. Although not yet indicative of fully formed clinical reasoning, these reflections represent early signs of higher-level empathy—characterized not only by emotional resonance but also by cognitive and ethical engagement with others’ lived experiences.

These observations are in line with previous studies that found role-playing can provoke negative emotional reactions among medical students. Some have cited role-play as their least preferred learning method due to discomfort, anxiety, or resistance tied to specific roles and expectations [45,46]. Practitioners may also hesitate due to the perceived artificiality of the exercise or discomfort under peer observation [47,48]. Such responses are often attributed to a lack of authenticity in simulated scenarios [17]. Similarly, disability simulations—which aim to cultivate empathy by temporarily restricting students’ vision, mobility, or sensory capacities—have been widely debated in the literature [22,49,50]. While some educators argue that these activities may raise awareness and foster short-term empathic concern [51], systematic reviews and empirical studies suggest that their effects on attitudes are minimal or inconsistent, and that they may even reinforce pity or negative stereotypes rather than genuine empathy [21,22,23]. While these critiques highlight potential limitations of role-based simulations, our LIWC analysis and human ratings provide empirical support that, when carefully structured and ethically implemented, role-playing can enhance empathy and perspective-taking by helping medical students cultivate sensitivity to others’ experiences in emotionally charged contexts, thereby strengthening their emotional awareness and interpersonal connection [14,15].

### Role-playing promotes elaborative thinking about communication

Our results showed that students in the caregiver role used more causation words, reflecting their effort to articulate and process the cause-and-effect dynamics inherent in caregiving. Providing care requires understanding the consequences of one’s actions and anticipating potential outcomes—skills that may be reinforced through the use of causal language. This aligns with findings by Pennebaker et al. [52], who noted a link between causation word use and improved expressive processing. Faced with the challenges of caregiving, students may rely on causal reasoning to manage uncertainty and complexity, which is reflected in their increased use of causation words.

Participants exhibited more discrepancy words in two contexts: during the Imagination Stage and when acting as caregivers. These words, which reflect a perceived gap between reality and expectations [26,28], appeared as students anticipated challenges, reflected on unmet goals, or considered future interactions. In the caregiver role, such language likely emerged in response to the emotional and practical complexities of addressing patients’ needs. During the Imagination Stage, students may have projected possible difficulties they would encounter, even before actual role-play began. After the Role-Playing Stage, there was a noticeable decline in discrepancy words and an increase in certainty words. This shift suggests a cognitive transition from anticipating uncertainties to confidently integrating and articulating their experiences [28].

Together, the increased use of these cognitive words reveals a progression in students’ reflective thinking—from anticipating challenges to integrating experiences with greater clarity. This cognitive shift highlights how role-playing not only evokes emotional responses but also deepens understanding of caregiving through intentional and empathetic reasoning [18].

## Limitations

Our study contributes to the use of language analysis in medical education, but there are limitations to consider. First, LIWC is a basic tool that does not account for context or content, and its interpretation (e.g., “I” as a marker of self-focus) relies on theoretical assumptions. While such indicators can be predictive [32], it is important to distinguish empirical findings from interpretive claims. In our data, the pronoun “I” often co-occurred with negative emotion words, a pattern likely shaped by the emotionally intense role-play context. Therefore, linguistic patterns should be interpreted within the situational and emotional context, not as isolated indicators of psychological states. Future research may benefit from combining word count data with qualitative content analysis. The second limitation is that the role-playing exercise, as a proxy for real communication, does not fully replicate real-life scenarios. Students participated in a class activity, pretending to be patients and caregivers, so our findings may not apply to real-world situations.

## Conclusion

Using the LIWC, we analyzed students’ reflective writing from both the Imagination and Role-Playing Stages to explore what they learned during role-playing. The analysis of linguistic, affective, and cognitive words revealed how students internalized their roles as patients and caregivers, reflected in their distinct language use in each context. This also led to more in-depth reflections on their emotions and cognition. The findings suggest that role-playing effectively encouraged students’ engagement in empathic communication and fostered a shift towards a more other-focused perspective.

In conclusion, language use provides valuable insights into the impact of role-playing on empathy and perspective-taking in communication courses. This serves as a useful tool for educators to assess students’ growth in learning communication. These findings have important implications for healthcare education, as embodying patient and caregiver roles helps students better understand patient needs and develop strategies for providing improved care.

## Declaration of generative AI and AI-assisted technologies in the writing process

During the preparation of this work, the author(s) used ChatGPT (OpenAI) to improve language and readability. After using this tool, the author(s) reviewed and edited the content as needed and take full responsibility for the content of the publication.

## Additional File

The additional file for this article can be found as follows:

10.5334/pme.1482.s1 Supplementary File. Supplementary Tables 1 to 4.
