# Supplementary figures and images for: Investigating the Influence of Role-Playing on Empathy and Perspective-Taking by Analyzing Level of Engagement, Emotional, and Cognitive Processes Through a Word Count Analysis Approach
Source: Perspect Med Educ. 2025 Nov 10;14(1):736–49. doi: 10.5334/pme.1482 (PMC12617402; doi:10.5334/pme.1482)

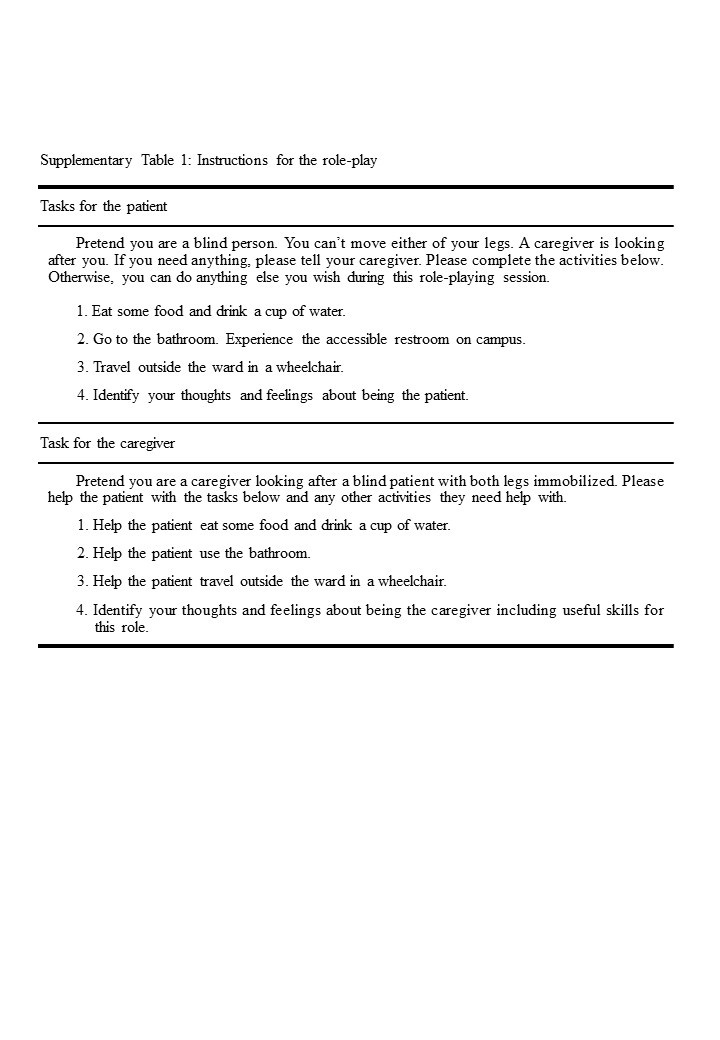

Supplement: Supplementary File. — Supplementary Tables 1 to 4. [file pme-14-1-1482-s1.zip › pme-1482_hsu-s1/6800bb692cd9b.jpg]

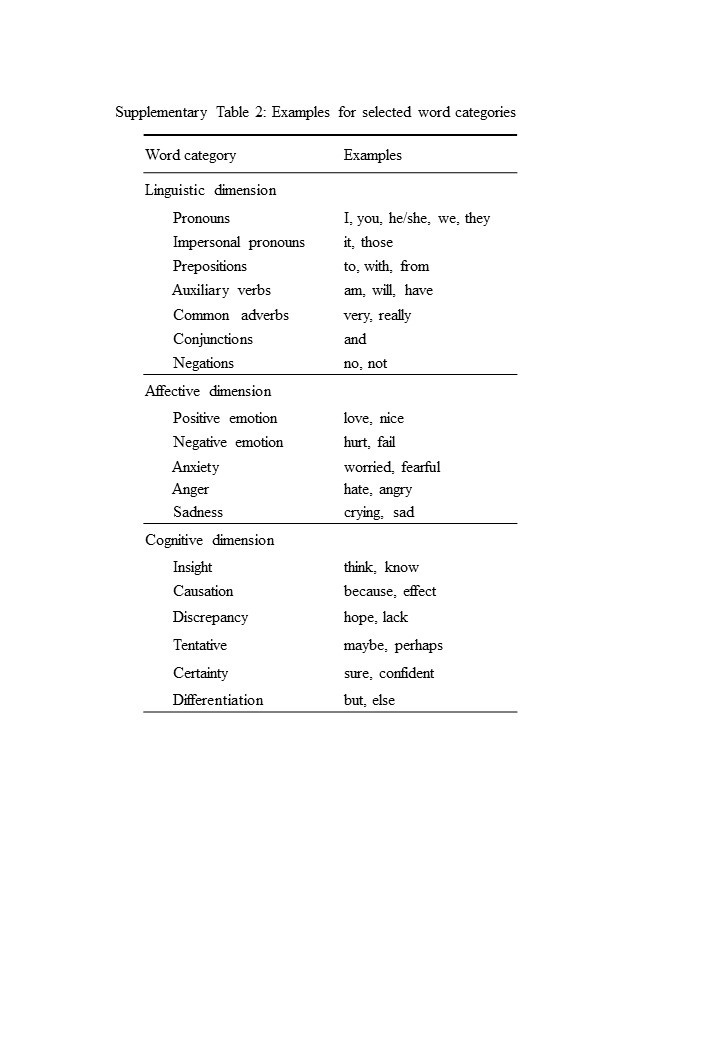

Supplement: Supplementary File. — Supplementary Tables 1 to 4. [file pme-14-1-1482-s1.zip › pme-1482_hsu-s1/6800bb91b9777.jpg]

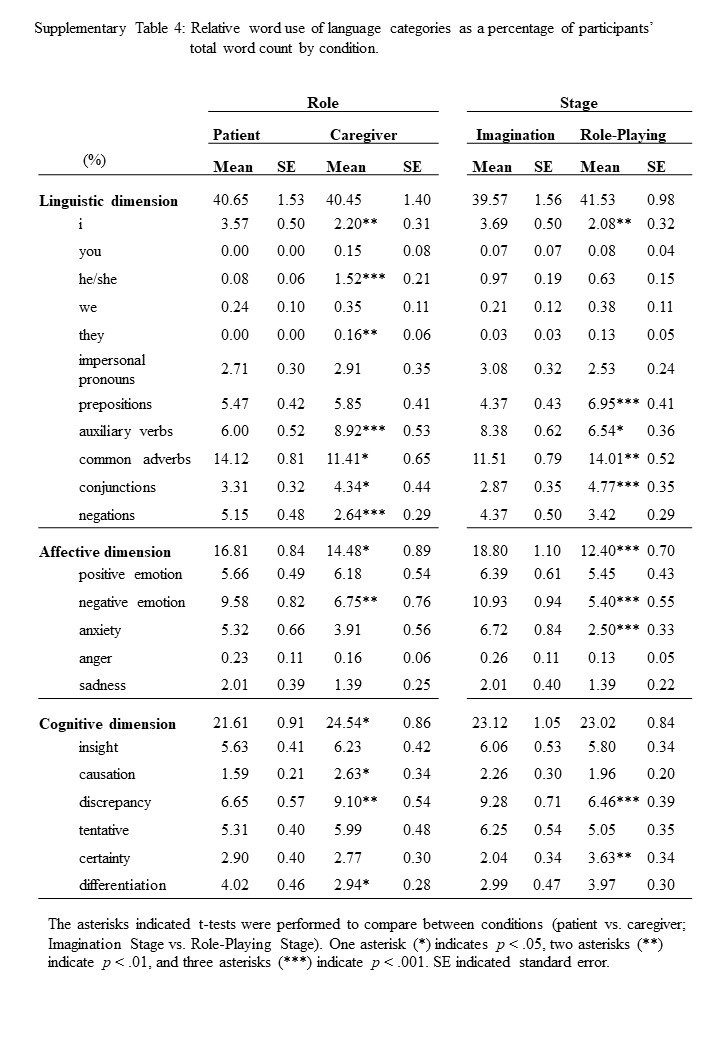

Supplement: Supplementary File. — Supplementary Tables 1 to 4. [file pme-14-1-1482-s1.zip › pme-1482_hsu-s1/6800bbe455f01.jpg]

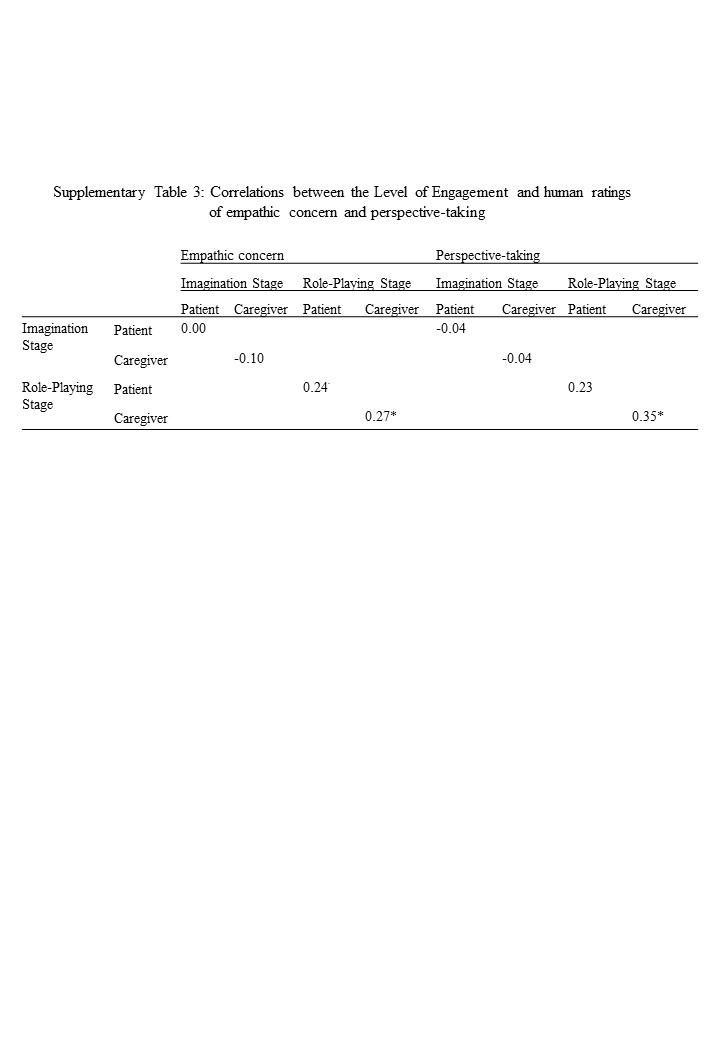

Supplement: Supplementary File. — Supplementary Tables 1 to 4. [file pme-14-1-1482-s1.zip › pme-1482_hsu-s1/680dd9819d5e1.jpg]
